# Supplementary material for: IFN-γ Induces PD-L1 Expression in Primed Human Basophils
Source: Cells. 2022 Feb 25;11(5):801. doi: 10.3390/cells11050801 (PMC8909048; doi:10.3390/cells11050801)
Supplement: Supplementary file 1 [file cells-11-00801-s001.zip › cells-1588050-supplementary.pdf]

cells

Article

IFN-γ Induces PD-L1 Expression in Primed Human Basophils

Srinivasa Reddy Bonam [1], Camille Chauvin [1], Mano J. Mathew [2], and Jagadeesh Bayry [1,3,*]

[1] Institut National de la Santé et de la Recherche Médicale, Centre de Recherche des Cordeliers, Sorbonne Université, Université de Paris, 75006 Paris, France.
[2] EFREI, Villejuif, 94800, France
[3] Department of Biological Sciences & Engineering, Indian Institute of Technology Palakkad, Palakkad, 678623, India.
* Correspondence: Jagadeesh.bayry@crc.jussieu.fr or bayry@iitpkd.ac.in

Citation: IFN-γ Induces PD-L1 Expression in Primed Human Basophils. *Cells* 2022, *11*, 801. https://doi.org/10.3390/cells11050801

Academic Editor(s):

Received: date
Accepted: date
Published: date

Publisher's Note: MDPI stays neutral with regard to jurisdictional claims in published maps and institutional affiliations.

Copyright: © 2022 by the authors. Submitted for possible open access publication under the terms and conditions of the Creative Commons Attribution (CC BY) license (https://creativecommons.org/licenses/by/4.0/).

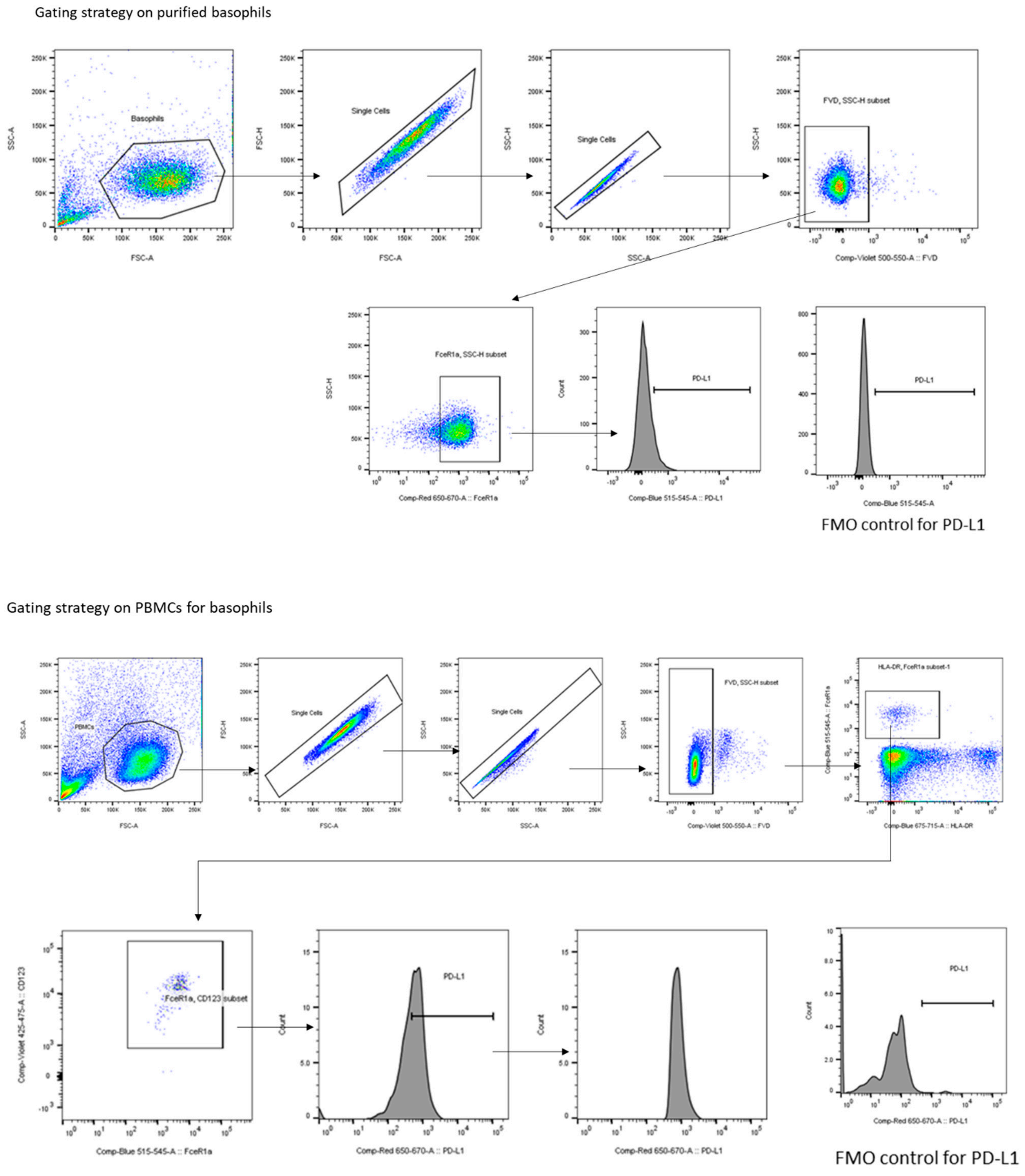

Figure S1. Flow cytometry gating strategy for the purified basophils (upper panels) and peripheral blood mononuclear cells containing basophils (lower panels).

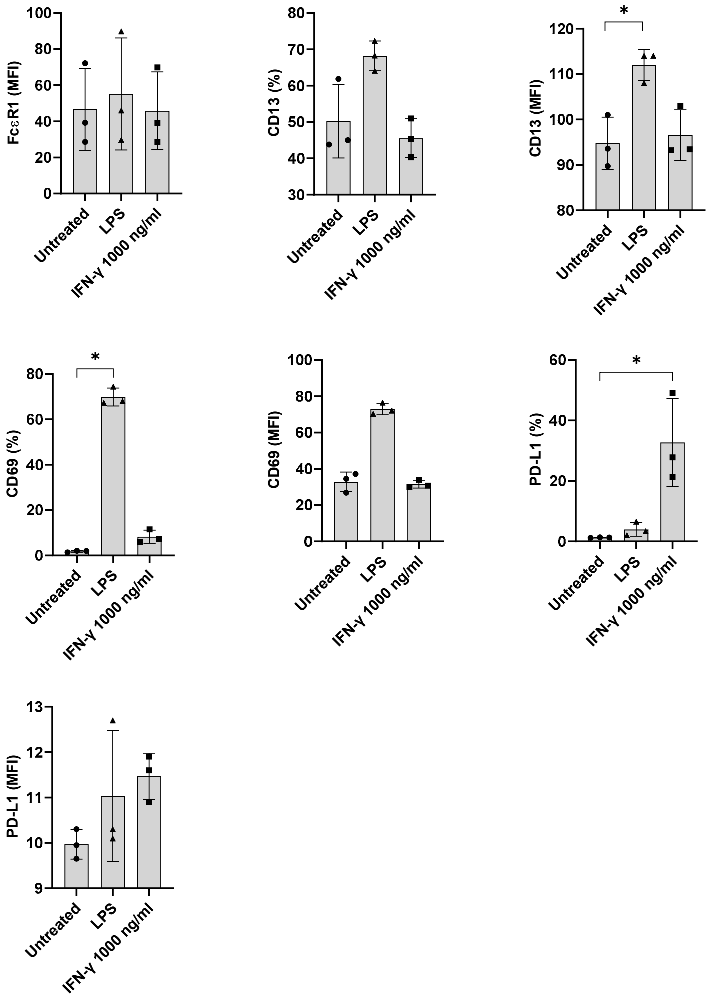

Figure S2. Prolonged stimulatory effect of IFN-γ on PD-L1 and other surface markers of untouched basophils. Basophil-containing PBMCs (1x10^{6} cells/ml/24-well plate) of healthy donors were cultured with or without IFN-γ for measuring different parameters. PBMCs were cultured either without (untreated) or with LPS (100 ng/ml) or with IFN-γ (1000 ng/ml) for 48 h. After incubation, PD-L1 expression (% positive cells and median fluorescence intensities (MFI), mean ± SD; n = 3 independent donors from two independent experiments) was evaluated by flow cytometry. *P < 0.05, one-way ANOVA Friedman test with Dunn's multiple comparisons post-test.

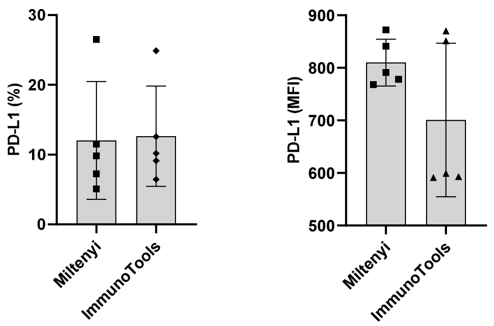

Figure S3. The effect of IFN-γ from different sources on PD-L1 expression in human basophils. Basophils (0.1x10^{6} cells/200 μl/96-well plate) isolated from PBMCs of healthy donors were cultured with IFN-γ at 1000 ng/ml. Basophil phenotype was evaluated by flow cytometry after 24 h. Expression of PD-L1 on basophils (% positive cells and median fluorescence intensities (MFI), mean ± SD; n = 5 independent donors from three independent experiments) was represented.

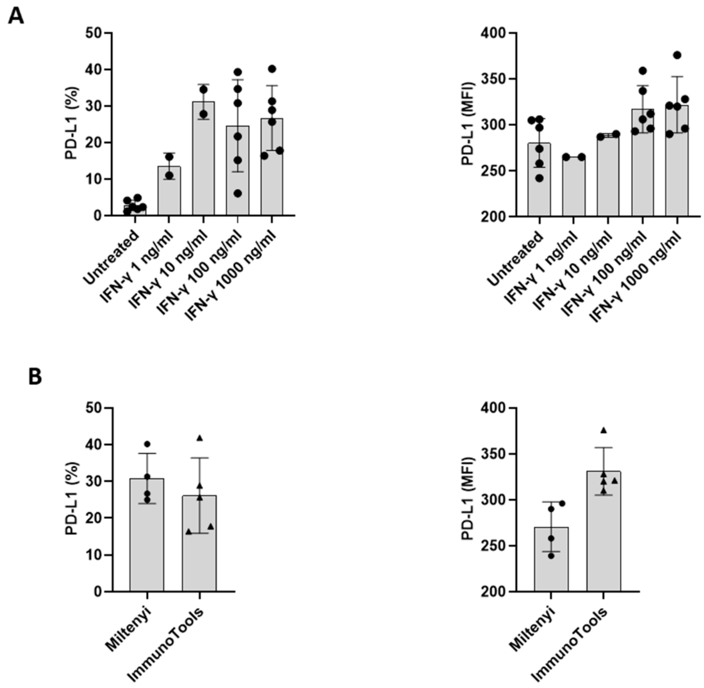

Figure S4. The influence of dose and source of IFN-γ towards PD-L1 expression in untouched basophils. Basophil-containing PBMCs (1x10[6] cells/ml/24-well plate) of healthy donors were cultured with or without IFN-γ for measuring different parameters. (A) PBMCs were cultured either without (untreated) or with IFN-γ at 1-1000 ng/ml for 24 h. After incubation, PD-L1 expression (% positive cells and median fluorescence intensities (MFI), mean ± SD; n = 2-6 independent donors from two or three independent experiments) was evaluated by flow cytometry. (B) PBMCs were treated with IFN-γ at 1000 ng/ml, from different sources, for 24 h. Expression of PD-L1 on basophils (% positive cells and median fluorescence intensities (MFI), mean ± SD; n = 4 independent donors from two independent experiments) was presented.

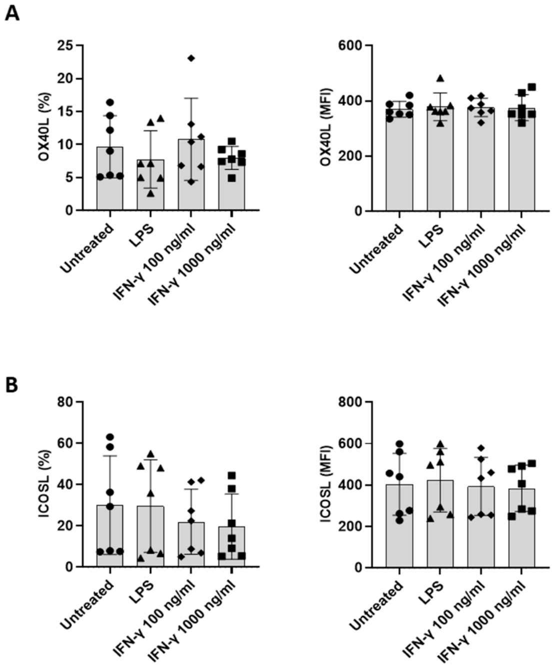

Figure S5. The effect of IFN-γ on OX40L and ICOSL expression in basophils from untouched PBMCs. Basophil-containing PBMCs (1x10[6] cells/ml/24-well plate) from the healthy donors were cultured with or without IFN-γ at 100 ng/ml and 1000 ng/ml or LPS at 100 ng/ml for 24 h. After incubation, cells phenotype was evaluated by flow cytometry. Expression of OX40L (A) and ICOSL (B) on the basophils (% positive cells and median fluorescence intensities (MFI), mean ± SD; n = 7 independent donors with three independent experiments) are presented.
